# The assembled transcriptome of the adult horn fly, *Haematobia irritans*

**DOI:** 10.1016/j.dib.2018.06.095

**Published:** 2018-07-02

**Authors:** Luisa N. Domingues, Felix D. Guerrero, Connor Cameron, Andrew Farmer, Kylie G. Bendele, Lane D. Foil

**Affiliations:** a USDA-ARS Knipling-Bushland, U. S. Livestock Insects Research Lab, Veterinary Pest Genomics Center, Kerrville, TX, USA; b National Center for Genome Resources, Santa Fe, NM, USA; c Department of Entomology, Louisiana State University, Baton Rouge, LA, USA

**Keywords:** Haematobia irritans irritans, RNAseq, Transcriptome, de novo assembly

## Abstract

The horn fly, *Haematobia irritans irritans* (Linnaeus, 1758; Diptera: Muscidae), a hematophagous external parasite of cattle, causes considerable economic losses to the livestock industry worldwide. This pest is mainly controlled with insecticides; however, horn fly populations from several countries have developed resistance to many of the products available for their control. In an attempt to better understand the adult horn fly and the development of resistance in natural populations, we used an Illumina paired-end read HiSeq and GAII approach to determine the transcriptomes of untreated control adult females, untreated control adult males, permethrin-treated surviving adult males and permethrin + piperonyl butoxide-treated killed adult males from a Louisiana population of horn flies with a moderate level of pyrethroid resistance. A total of 128,769,829, 127,276,458, 67,653,920, and 64,270,124 quality-filtered Illumina reads were obtained for untreated control adult females, untreated control adult males, permethrin-treated surviving adult males and permethrin + piperonyl butoxide-treated killed adult males, respectively. The *de novo* assemblies using CLC Genomics Workbench 8.0.1 yielded 15,699, 11,961, 2672, 7278 contigs (≥ 200 nt) for untreated control adult females, untreated control adult males, permethrin-treated surviving adult males and permethrin + piperonyl butoxide-treated killed adult males, respectively. More than 56% of the assembled contigs of each data set had significant hits in the BlastX (UniProtKB/Swiss-Prot database) (E <0.001). The number of contigs in each data set with InterProScan, GO mapping, Enzyme codes and KEGG pathway annotations were: Untreated Control Adult Females – 10,331, 8770, 2963, 2183; Untreated control adult males – 8392, 7056, 2449, 1765; Permethrin-treated surviving adult males – 1992, 1609, 641, 495; Permethrin + PBO-treated killed adult males – 5561, 4463, 1628, 1211.

**Specifications Table**

**Table:** 

Subject area	Biology
More specific subject area	Insect transcriptome
Type of data	Transcriptome sequences and associated annotations (tables, text file)
How data was acquired	2×54 paired-end read RNAseq of RNA isolated from whole newly emerged unfed adult flies
Data format	Raw FASTQ and processed FASTA sequence files, including assembled transcriptome FASTA files
Experimental factors	Isolates: Newly emerged unfed adult females, newly emerged unfed adult males, newly emerged unfed adult males treated with permethrin, newly emerged unfed adult males treated with permethrin + piperonyl butoxide
Experimental features	Assembled transcriptomes of whole body of newly emerged unfed adult flies (Untreated Control Adult Females, Untreated Control Adult Males, Permethrin-Treated Surviving Adult Males and Permethrin + Piperonyl Butoxide-Treated Killed Adult Males)
Data source location	St. Gabriel, Louisiana, USA
Data accessibility	Data is with this article and also available at the National Center for Biotechnology Information (NCBI) Short Read Archive (SRA) through the direct link https://www.ncbi.nlm.nih.gov/sra/SRP131897 or through SRA accession number SRP131897. The adult horn fly transcriptome Shotgun Assembly project has been deposited at DDBJ/EMBL/GenBank under the accession GGLM00000000. The version described in this paper is the first version, GGLM01000000. The overall BioProject ID is PRJNA429442 and the BioSample accessions are SAMN08355023, SAMN08355024, SAMN08355025, and SAMN08355026.

**Value of the data**

• Resource for investigations of the molecular basis of insecticide resistance in the horn fly, *Haematobia irritans irritans.*
• Provides candidate protein coding regions for the development of control strategies targeting adult flies.

## 1 Data

RNA was isolated from unfed, newly emerged adult horn flies, including untreated control adult females, untreated control adult males, permethrin-treated surviving adult males and permethrin + piperonyl butoxide-treated killed adult males. Subsequently, a single lane of 2 × 54 bp paired end RNASeq reads were obtained, de novo assembled and annotated. The raw reads are accessible at NCBI׳s SRA through the direct link https://www.ncbi.nlm.nih.gov/sra/SRP131897 or through SRA accession number SRP131897. The adult horn fly transcriptome Shotgun Assembly project has been deposited at DDBJ/EMBL/GenBank under the accession GGLM00000000. The version described in this paper is the first version, GGLM01000000. The overall BioProject ID is PRJNA429442 and the BioSample accessions are SAMN08355023, SAMN08355024, SAMN08355025, and SAMN08355026.

## 2 Experimental design, materials and methods

### 2.1 Flies

Adult flies were collected with aerial sweep hand nets from pastured cattle at the St. Gabriel Research Station, Saint Gabriel, Louisiana, and incubated in large inverted Erlenmeyer flasks to collect eggs that were immediately seeded into manure to allow adult fly emergence. The unfed, newly emerged flies were sexed and either immediately frozen at −80 °C for sequencing (females and males) or exposed to low doses of permethrin (1.56 µg/cm^2^, ~LD25) or permethrin (1.56 µg/cm^2^, ~LD25) + 1% piperonyl butoxide (PBO) by the impregnated filter paper assay [1] for 2 h. Adult male flies that survived exposure to permethrin and adult male flies killed by exposure to permethrin +PBO were frozen at −80 °C for sequencing.

### 2.2 RNA isolation

Fourteen unfed, newly emerged adult flies from the untreated control females, untreated control males, permethrin-treated males and permethrin + PBO-treated males groups were used to purify total RNA in a protocol adapted for use with the FastPrep 24 Tissue and Cell Homogeneizer (MP Biomedicals, Solon, OH, USA) and the FastRNA Pro Green Kit (MP Biomedicals).

### 2.3 Sequencing and bioinformatics

Sequencing was performed at the National Center for Genome Research (Santa Fe, NM, USA) using the standard Illumina RNAseq library preparation protocol and a single lane of the RNAseq. 2 × 54 paired-end approach. A total of 134,671,818, 132,374,494, 68,856,572, 65,427,160 paired-end Illumina raw reads were produced for untreated control adult females, untreated control adult males, permethrin-treated surviving adult males and permethrin + PBO-treated killed adult males, respectively (Table 1). The raw reads of all four datasets were trimmed using either CLC Genomics Workbench 8.0.1 (https://www.qiagenbioinformatics.com/) or Trimmomatic programmable-0.33 [2] (https://de.cyverse.org/de/?type=apps&app-id=8cb5c088-6b3e-11e7-a22d-008cfa5ae621&system-id=de) (parameters:  SLIDINGWINDOW: 4:20, LEADING: 3, TRAILING: 3, MINLEN: 20) followed by Sickle-quality-based-trimming_version_1.0  [3]  (https://de.cyverse.org/de/?type=apps&app-id=9f5710c6-3424-11e7-9a58-008cfa5ae621&system-id=de) (parameters: quality threshold 20, minimum length 20) and Illumina adaptor sequences and low quality bases were removed. Trimmomatic and Sickle were both run on CyVerse/Discovery Environment [4]. The raw reads were assembled with three assemblers for comparison: CLC Genomics  Workbench  8.0.1,  Trinity  version  2.5.1  [5]  (https://de.cyverse.org/de/?type=apps&app-id=trinity-wrangler-2.5.1u2&system-id=agave) or version 11.10.13 (https://de.cyverse.org/de/?type=apps&app-id=trinity-stmpde-11.10.13u2&system-id=agave) and SoapdenovoTrans version 1.0.3 [6] (https://de.cyverse.org/de/?type=apps&app-id=Soaptrans-1.0.3u1&system-id=agave). Both Trinity versions and SoapdenovoTrans were run on CyVerse/Discovery Environment [4]. The kmer lengths used were 21, 23, 24, 25, 27, 29, 31, 32 and 33 for CLC Genomics Workbench 8.0.1, 21, 23, 25, 27, 29, 31, 32 for Trinity version 2.5.1, 25 for Trinity version 11.10.13, and 21, 25, 27, 29, 33 for SoapdenovoTrans version 1.0.3 (Supplementary Table 1).

**Table 1:** Trim strategy, assembler, kmer length and summarized BUSCO annotation for the best assemblies. Results for all assemblies performed can be seen at Supplementary Table 1.

Datasets	Trim	Assembler	Kmer length	Summarized benchmarking in BUSCO annotation**
Untreated Control Adult Females	Trimmomatic/Sickle*	CLC Genomics Workbench 8.0.1	21	C:44.2%[S:43.8%,D:0.4%],
				F:14.9%,M:40.9%
Untreated Control Adult Males	CLC Genomics Workbench 8.0.1	CLC Genomics Workbench 8.0.1	21	C:26.5%[S:26.4%,D:0.1%],
				F:13.6%,M:59.9%
Permethrin-Treated Surviving Adult Males	CLC Genomics Workbench 8.0.1	CLC Genomics Workbench 8.0.1	21	C:2.8%[S:2.8%,D:0.0%],
				F:2.3%,M:94.9%
Permethrin + PBO-Treated Killed Adult Males	Trimmomatic/Sickle	CLC Genomics Workbench 8.0.1	21	C:9.8%[S:9.8%,D:0.0%],
				F:11.1%,M:79.1%

* Trimmomatic  programmable-0.33  [2]  (https://de.cyverse.org/de/?type=apps&app-id=8cb5c088-6b3e-11e7-a22d-008cfa5ae621&system-id=de) (parameters: SLIDINGWINDOW: 4:20, LEADING: 3, TRAILING: 3, MINLEN: 20). Sickle-quality-based-trimmimg_version_1.0  [3]  (https://de.cyverse.org/de/?type=apps&app-id=9f5710c6-3424-11e7-9a58-008cfa5ae621&system-id=de).

** BUSCO version 3.0.2 [8]. Lineage dataset: diptera_odb9 (Creation date: 2016-10-21, number of species: 25, number of BUSCOs: 2799). BUSCO was run in mode: transcriptome. C: Complete BUSCOs, S: Complete and single-copy BUSCOs, D: Complete and duplicated BUSCOs, F: Fragmented BUSCOs; M: Missing BUSCOs.

The assembled transcriptomes were then compared using three tools on CyVerse/Discovery Environment [4]: Compute Contig Statistics (https://pods.iplantcollaborative.org/wiki/display/DEapps/Compute+Contig+Statistics), rnaQUAST_1.2.0 (de novo based) [7] (https://de.cyverse.org/de/?type=apps&app-id=980dd11a-1666-11e6-9122-930ba8f23352&system-id=de) and BUSCO-v3.0  [8]  (https://de.cyverse.org/de/?type=apps&app-id=7f948668-7a53-11e7-a680-008cfa5ae621&system-id=de) (Supplementary Table 1). Assemblies with the lowest percentage of missing BUSCOs were considered the best (Table 1) and were submitted to the NCBI Transcriptome Shotgun Assembly (TSA) database after screening with the NCBI foreign contamination screen protocol. Supplementary Files 1–4 contain the FastA sequences of the final assembled database for untreated control adult females (15,699 entries > 200 nt), untreated control adult males (11,961 entries > 200 nt), permethrin-treated surviving adult males (2672 entries > 200 nt), and permethrin + PBO-treated killed adult males (7278 entries > 200 nt), respectively.

**Fig. 1:**
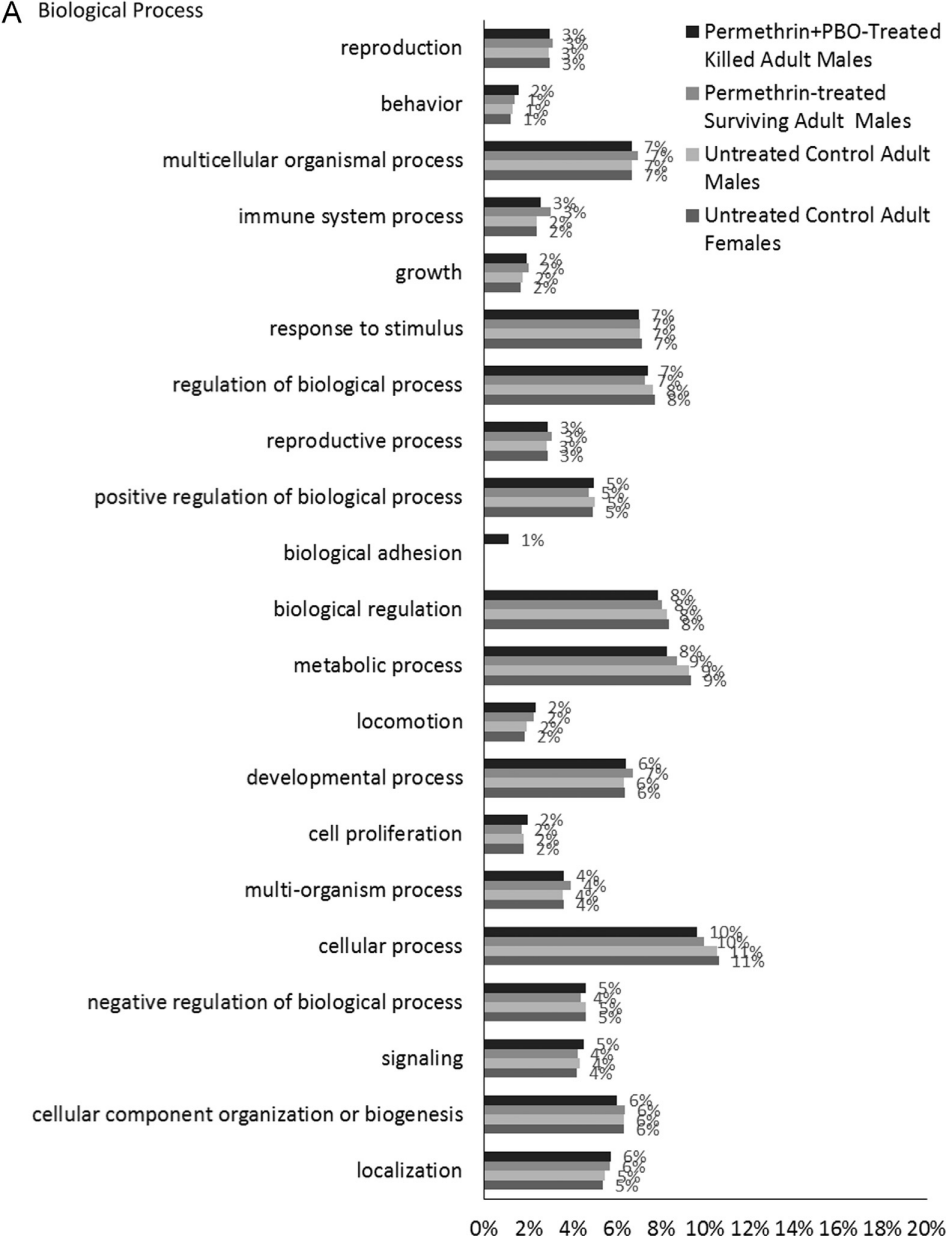

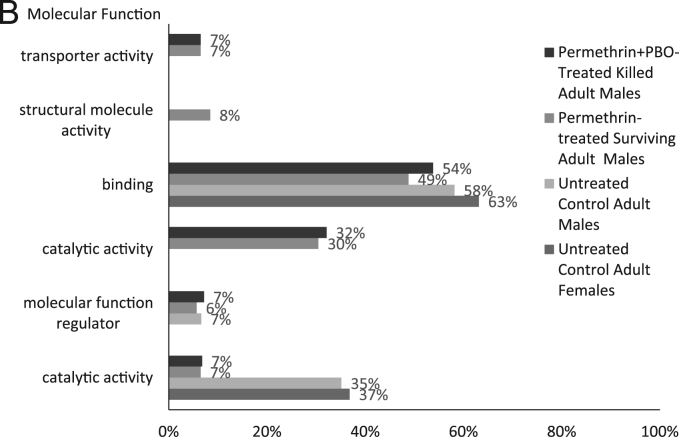

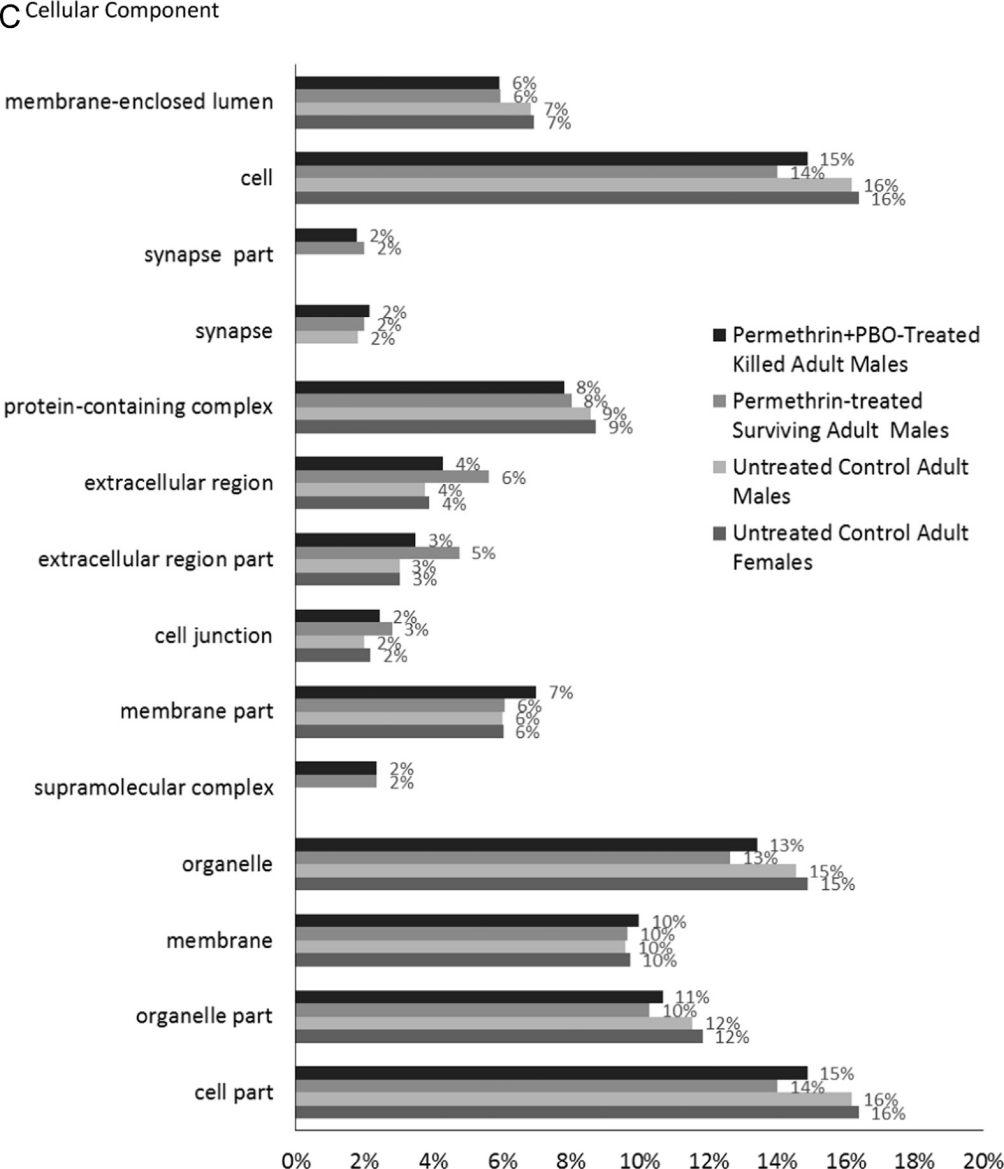
Gene Ontology Classifications of assembled transcriptomes. All four transcriptomes were annotated with Blast2GO PRO (version 5.0.21) mapping and level 2 GO terms for Biological Process (A), Molecular Function (B) and Cellular Component (C) ontologies. The percentage of annotated transcripts with each indicated GO term level 2 is shown.

The transcriptomes were BlastX aligned against the UniProtKB/SwissProt database (E-value = 1.0 e^-3^) using Blast2GO PRO version 5.0.21 [9], [10], [11], [12], and annotated using Blast2GO Pro GO Annotation and InterProScan performed using Blast2GO PRO Annotation. KEGG Pathway maps were determined using Blast2GO PRO version 5.0.21 [13]. Statistics of the transcriptomes can be seen in Table 2. Fig. 1 shows the functional annotation of the four transcriptomes for Gene Ontology Level 2 Terms for Biological Process, Molecular Function and Cellular Component. Detailed transcript annotation including BlastX hits, GO terms, InterProScan, Enzyme Codes and KEGG Pathway data can be found in Supplementary Tables 2–5.

**Table 2:** Statistics of transcriptomes assembled on CLC Genomics Workbench 8.0.1 using the following parameters: kmer size = 21, minimum contig length of 200 and mapping options as default (mismatch cost: 2, insertion cost: 3, deletion cost: 3, length fraction: 0.5, similarity fraction: 0.8).

Parameters	Untreated Control Adult Females	Untreated Control Adult Males	Permethrin-Treated Surviving Adult Males	Permethrin + PBO-Treated Killed Adult Males
Number of raw reads	134,671,818	132,374,494	68,856,572	65,427,160
Number of raw reads post trimming	126,531,116	127,276,458	66,325,598	61,732,240
Number of contigs (>200 nt)	15,699	11,961	2672	7278
Total size of contigs (nt)	15,836,681	10,217,787	2,048,521	6,733,471
Longest contig (nt)	20,187	21,272	16,352	33,914
Average contig length (nt)	1,009	854	767	925
N50 (nt)	1607	1267	988	1341
Number of contigs > 500 bp (%)	9331 (59%)	6452 (54%)	1264 (47%)	4094 (56%)
Number of contigs > 1000 bp (%)	5386 (34%)	3136 (26%)	477 (18%)	1922 (26%)
Contigs with BlastX hits (%)	8778 (56%)	7064 (59%)	1609 (60%)	4472 (61%)
Contigs with InterProScan (%)	10,331 (66%)	8392 (70%)	1992 (75%)	5561 (76%)
Contigs with GO Mapping (%)	8770 (56%)	7056 (59%)	1609 (60%)	4463 (61%)
Contigs with Enzyme Codes (%)	2963 (19%)	2449 (20%)	641 (24%)	1628 (22%)
Contigs with KEGG Pathway (%)	2183 (14%)	1765 (15%)	495 (19%)	1211 (17%)

## References

[1] Sheppard D.C., Hinkle N.C. (1987). A field procedure using disposable materials to evaluate horn fly insecticide resistance. J. Agric. Entomol..

[2] Bolger A.M., Lohse M., Usadel B. (2014). Trimmomatic: a flexible trimmer for Illumina Sequence Data. Bioinformatics.

[3] N.A. Joshi, J.N. Fass, Sickle: A sliding-window, adaptive, quality-based trimming tool for FastQ files (Version 1.33) [Software]. (2011) Available at 〈https://github.com/najoshi/sickle〉.

[4] Merchant N., Lyons E., Goff S., Vaughn M., Ware D., Micklos D., Antin P. (2016). The iPlant Collaborative: cyber infrastructure for enabling data to discovery for the life sciences. PLoS Biol..

[5] Grabherr M.G., Haas B.J., Yassour M., Levin J.Z., Thompson D.A., Amit I., Adiconis X., Fan L., Raychowdhury R., Zeng Q., Chen Z., Mauceli E., Hacohen N., Gnirke A., Rhind N., di Palma F., Birren B.W., Nusbaum C., Lindblad-Toh K., Friedman N., Regev A. (2011). Full-length transcriptome assembly from RNA-seq data without a reference genome. Nat. Biotechnol..

[6] Xie Y., Wu G., Tang J., Luo R., Patterson J., Liu S., Huang W., He G., Gu S., Li S., Zhou X., Lam T., Li Y., Xu X., Ka-Shu Wong G., Wang J. (2014). SOAPdenovo-Trans: de novo transcriptome assembly with short RNA-Seq reads. Bioinformatics.

[7] Bushmanova E., Antipov D., Lapidus A., Suvorov V., Prjibelski A.D. (2016). rnaQUAST: a quality assessment tool for de novo transcriptome assemblies. Bioinformatics.

[8] Simao F.A., Waterhouse R.M., Ioannidis P., Kriventseva E.V., Zdobnov E.M. (2015). BUSCO: assessing genome assembly and annotation completeness with single-copy orthologs. Bioinformatics.

[9] Conesa A., Götz S., Garcia-Gomez J.M., Terol J., Talon M., Robles M. (2005). Blast2GO: a universal tool for annotation, visualization and analysis in functional genomics research. Bioinformatics.

[10] Conesa A., Götz S. (2008). Blast2GO: a comprehensive suite for functional analysis in plant genomics. Int. J. Plant Genom..

[11] Götz S., García-Gómez J.M., Terol J., Williams T.D., Nagaraj S.H., Nueda M.J., Robles M., Talón M., Dopazo J., Conesa A. (2008). High-throughput functional annotation and data mining with the Blast2GO suite. Nucleic Acids Res..

[12] Götz S., Arnold R., Sebastián-León P., Martín-Rodríguez S., Tischler P., Jehl Marc-André, Dopazo J., Rattei T., Conesa A. (2011). B2G-FAR, a species centered GO annotation repository. Bioinformatics.

[13] Kanehisa M., Goto S. (2000). KEGG: kyoto encyclopedia of genes and genomes. Nucleic Acids Re.s.

